# Geriatric care for surgical patients: results and reflections from a cross-sectional survey in acute Belgian hospitals

**DOI:** 10.1007/s41999-023-00748-3

**Published:** 2023-01-24

**Authors:** Katleen Fagard, Mieke Deschodt, Lisa Geyskens, Sarah Willems, Benoît Boland, Albert Wolthuis, Johan Flamaing

**Affiliations:** 1 Department of Geriatric Medicine, University Hospitals Leuven, Dienst Geriatrie UZ Leuven, Herestraat 49, Box 7003 35, 3000 Leuven, Belgium; 2 Department of Public Health and Primary Care, KU Leuven, Leuven, Belgium; 3 Competence Centre of Nursing, University Hospitals Leuven, Leuven, Belgium; 4 Department of Geriatric Medicine, University Hospital Saint-Luc, Brussels, Belgium; 5 Department of Abdominal Surgery, University Hospitals Leuven, Leuven, Belgium

**Keywords:** Older adults, Perioperative medicine, Geriatric co-management, Geriatric surgery, Survey

## Abstract

**Purpose:**

To explore geriatric care for surgical patients in Belgian hospitals and geriatricians’ reflections on current practice.

**Methods:**

A web-based survey was developed based on literature review and local expertise, and was pretested with 4 participants. In June 2021, the 27-question survey was sent to 91 heads of geriatrics departments. Descriptive statistics and thematic analysis were performed.

**Results:**

Fifty-four surveys were completed, corresponding to a response rate of 59%. Preoperative geriatric risk screening is performed in 25 hospitals and systematically followed by geriatric assessment in 17 hospitals. During the perioperative hospitalisation, 91% of geriatric teams provide non-medical and 82% provide medical advice. To a lesser extent, they provide geriatric protocols, geriatric education and training, and attend multidisciplinary team meetings. Overall, time allocation of geriatric teams goes mainly to postoperative evaluations and interventions, rather than to preoperative assessment and care planning. Most surgical patients are hospitalised on surgical wards, with reactive (73%) or proactive (46%) geriatric consultation. In 36 hospitals, surgical patients are also admitted on geriatric wards, predominantly orthopaedic/trauma, abdominal and vascular surgery. Ninety-eight per cent of geriatricians feel that more geriatric input for surgical patients is needed. The most common reported barriers to further implement geriatric-surgical services are shortage of geriatricians and geriatric nurses, and unadjusted legislation and financing.

**Conclusion:**

Geriatric care for surgical patients in Belgian hospitals is mainly reactive, although geriatricians favour more proactive services. The main opportunities and challenges for improvement are to resolve staff shortages in the geriatric work field and to update legislation and financing.

**Supplementary Information:**

The online version contains supplementary material available at 10.1007/s41999-023-00748-3.

## Key summary points

**Aim:**

This survey aims to explore geriatric care for surgical patients in acute Belgian hospitals and geriatricians’ reflections on current practice.

**Findings:**

To date, geriatric support for surgical patients in Belgian hospitals is mainly reactive, although geriatricians favour more proactive care. The main barriers to expand geriatric support are shortage of geriatricians and geriatric nurses, lack of financing and inadequate legislation.

**Message:**

A transition towards proactive models of care and adapted financing is needed to improve geriatric support for older surgical patients.

**Supplementary Information:**

The online version contains supplementary material available at 10.1007/s41999-023-00748-3.

## Introduction

With increasing life expectancy and age-related comorbidities, the number of older patients referred for surgery is steadily rising [1, 2]. Consequently, surgical teams are faced with a growing number of patients with frailty, multimorbidity, polypharmacy and complex needs in terms of medical, mental, functional, social and nutritional support. Moreover, older patients are at higher risk of developing perioperative complications and geriatric syndromes [3, 4].

To deal with the complexity of older patients, acute geriatric hospital wards have become standard practice decades ago [5]. Key factors for their success are the multidimensional approach in which every patient receives a comprehensive geriatric assessment that leads to an individualised treatment plan and early rehabilitation [6]. Other important drivers are interdisciplinary collaboration, knowledge and experience within the team regarding common geriatric problems and involvement of patients and caregivers in determining care goals [7]. These wards mainly accommodate patients with acute medical problems, acute geriatric syndromes or rehabilitation needs. Admission of surgical patients on acute geriatric hospital wards is less common [8, 9].

In Belgium, geriatric expertise for older patients on non-geriatric wards, such as surgical wards, is mainly provided by mobile inpatient geriatric consultation teams (IGCT) that visit high-risk older patients upon request and recommend interventions to the responsible care team [10, 11]. Their effectiveness is limited because of the recommendation-based character, the rather reactive than proactive approach, the lack of geriatric knowledge and expertise in non-geriatric teams and a low adherence to the proposed interventions [11–13]. To overcome these barriers, research and clinical practice are shifting from the above-mentioned consultative model towards co-management models. Geriatric-surgical co-management is characterised by direct proactive care, collaboration and shared responsibility between the surgical and geriatric team [14]. A recent review, including 12 studies of which nine in an orthogeriatric population, indicated that geriatric-surgical co-management is likely to reduce length of stay, mortality and readmission rates, although the grade of evidence was low [15]. Apart from co-management on geriatric or surgery wards, preoperative outpatient clinics providing geriatric assessment and optimisation are also among the newer initiatives for older surgical candidates [16–18]. A recent review of their effectiveness found 3 randomised controlled trials and concluded that there is low grade evidence for reduced mortality up to 3 months and 30-day overall complication rate [18].

Previous surveys to map geriatric care for surgical patients, conducted in the UK (2013 and 2017) and in Australia/New Zealand (2018), demonstrated that reactive consultative services are still the predominant care model [19–21]. A lack of financing for more proactive geriatric care was the most perceived barrier to establish proactive care services. In Belgium, a Royal Decree issued in 2007 and updated in 2014 still recommends the consultative IGCT model on request of the surgical team [10]. IGCTs are financed by the Belgian healthcare system and therefore remain the predominant model of geriatric care for surgical patients [11]. Nevertheless, more proactive services, such as co-management initiatives during the perioperative hospitalisation and preoperative optimisation clinics, are emerging. However, their distribution among Belgian hospitals is not known.

The aim of the present survey is twofold: (1) to explore which geriatric services are provided for surgical patients in acute Belgian hospitals and (2) to explore geriatricians’ reflections on current practice.

## Methods

### Design and sample

A cross-sectional survey was conducted in all Belgian hospitals with a geriatrics and a surgery department. Eligible hospitals were identified through a website listing health institutions in Belgium [22]. Heads of geriatrics departments, to whom we sent the survey, were identified through the hospitals' websites.

### Survey development and validation

The survey questionnaire was developed based on literature review and the research team’s expertise in care model development and evaluation. The original survey was available in two languages: Dutch (designed and adapted by KF, LG, MD and JF) and French (translated by KF and BB). An English translation is available in Online Appendix 1. Closed-ended, multiple choice, ranking, Likert scale and open-ended questions were used.

To ensure readability, face and content validity, the questionnaire was first reviewed by a two experts in geriatric care models and then pretested in a small sample of four participants (see acknowledgements). The final version consisted of 27 questions (with subquestions according to the answer given), divided into three main sections: (1) geriatric services for surgical patients in the hospital, (2) reflections on current practice and ideas for the future and (3) general information about the hospital and the geriatrics department.

The first section included questions about the surgical specialties present in the hospital (1 question), preoperative geriatric screening and geriatric assessment (4 questions), perioperative services for surgical teams (7 questions), time allocation of geriatric teams to perioperative care (1 question) and geriatric-surgical care models for hospitalised surgical patients (1 question). We examined which of the following four geriatric-surgical care models were implemented in the hospital: (1) the patient is hospitalised on a surgical ward with geriatric consultation on request, (2) the patient is hospitalised on a surgical ward with proactive geriatric consultation from admission to discharge, (3) the patient is hospitalised on a geriatric ward with proactive surgical consultation from admission to discharge, and (4) the patient is hospitalised on a geriatric ward with surgical consultation on request. The surveyed models were initially described by Kammerlander et al. [23]. For each model, the surgical specialities to which the model is applied in the hospital had to be indicated, with the possibility of applying different models for one particular surgical specialty.

In the second section, we inquired about the preferred geriatric-surgical care model (1 question), the perceived need for geriatric input for surgical patients in the own hospital (2 questions) and barriers to further develop geriatric-surgical care in the own hospital (2 questions). We also provided the opportunity to formulate further comments or suggestions regarding perioperative care for geriatric patients in Belgium (1 question).

The third section included questions about the size of the hospital and the geriatrics department, and characteristics of the geriatrics department in terms of services and staffing (7 questions).

### Data collection procedure

The survey was built in REDCap (Research Electronic Data Capture). A web link to the online survey was sent to the head of the geriatrics department of 91 hospitals in June 2021. Between July and November 2021, up to three reminders were sent by e-mail. Non-responders were contacted by telephone. The survey was conducted in compliance with the principles of the Declaration of Helsinki (latest version 2013), the principles of Good Clinical Practice and General Data Protection Regulation and in accordance with all applicable regulatory requirements. The study did not need approval by the Ethics Committee. The participants participated on a voluntary basis, no sensitive patient data were collected, and the reporting of the results does not reveal the participants’ identity. No financial compensation for participation was provided.

### Data analysis

Descriptive statistics were used to summarise responses. Continuous variables were reported as medians with interquartile ranges and ranges. Categorical variables were reported as numbers and percentages. Data were analysed using SPSS for Windows version 27 (SPSS Inc., Chicago, IL, USA). Thematic analysis was used to identify key concepts and themes in the free-text responses. The thematic analysis was summarised narratively.

## Results

### Characteristics of responding hospitals

Fifty-four participants completed the survey, resulting in a response rate of 59%. Characteristics of responding hospitals are summarised in Table 1. The total number of operational beds on acute geriatric hospital wards (G-beds) varied from 24 to 330 per hospital, divided over 1 to 5 campuses. For every 24 operational G-beds, hospitals employ a median of 1.0 full-time equivalent (FTE) geriatrician and 0.6 FTE trainees in internal, geriatric or general medicine. Twenty-one hospitals (39%) employ general physicians to assist in the care for geriatric patients. An IGCT is present in every hospital. The median number of IGCT members (nurses and allied health professionals) per hospital is 2.9 FTE, ranging from 0.5 to 6.8. Eighteen hospitals (33%) employ an advanced practice nurse, i.e. a master's degree trained nurse responsible for the introduction and maintenance of hospital-wide innovative geriatric care. Abdominal, orthopaedic/trauma, vascular and urologic surgery were present in every hospital, whereas cardiac surgery was the least represented surgical specialty.

**Table 1:** Characteristics of responding hospitals (*n* = 54)

Characteristic	Result
Number of operational G-beds, median (IQR) (range)	72 (49–108) (24–330)
Number of campuses, *n* (%)	
1	31 (57)
2	11 (20)
3	9 (17)
4	2 (4)
5	1 (2)
Number of FTE geriatricians, median (IQR) (range)	
Per hospital	3.1 (2–5) (1–11)
Per 24 operational G-beds	1.0 (0.9–1.3) (0.4–2.7)
Number of FTE trainees in internal, geriatric or general medicine in the geriatrics department, median (IQR) (range)	
Per hospital	2.0 (1.0–4.3) (0–22)
Per 24 operational G-beds	0.6 (0.2–1.2) (0–5)
Number of hospitals with general physicians in the geriatrics department, *n* (%)	21 (39%)
Number of FTE general physicians in the geriatrics department, median (IQR) (range)	
Per hospital	0 (0–1) (0–7)
Per 24 operational G-beds	0 (0–0.4) (0–1.5)
Components of the care programme for geriatric patients, *n* (%)	
Acute geriatric hospital ward (G-beds)	54 (100)
Geriatric day hospital	54 (100)
IGCT	54 (100)
Geriatric outpatient clinic	53 (98)
External liaison service^a^	48 (89)
Number of FTE IGCT members (non-physicians), median (IQR) (range)	2.9 (1.5–4.0) (0.5–6.8)
Number of hospitals with an advanced practice nurse in geriatric care, *n* (%)	18 (33)
Surgical specialties in the hospital, *n* (%)	
Orthopaedic/trauma surgery	54 (100)
Abdominal surgery	54 (100)
Vascular surgery	54 (100)
Urologic surgery	54 (100)
Ear–nose–throat surgery	53 (98)
Ophthalmologic surgery	51 (94)
Gynaecologic/breast surgery	49 (91)
Plastic/reconstructive surgery	48 (89)
Maxillofacial surgery	44 (82)
Oncologic surgery	44 (82)
Thoracic surgery	41 (76)
Neurosurgery	38 (70)
Cardiac surgery	21 (39)

*FTE* full-time equivalent, *G* Geriatric, *IGCT* Internal geriatric consultation team, *IQR* Inter quartile range, *n* Number

^a^Focuses on developing partnerships between the hospital, home care services and external facilities, and on integrating transitional care programmes

### Geriatric services for surgical patients and surgical teams

**Fig. 1:**
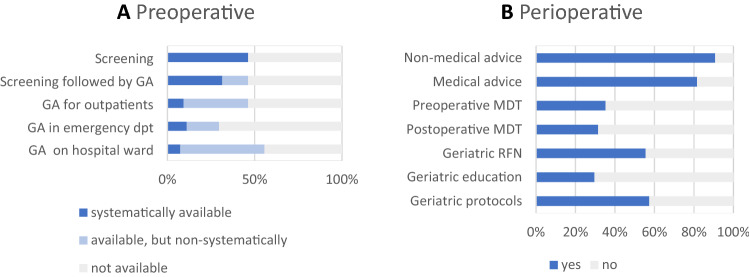
Geriatric services for surgical patients and surgical teams. **A** Preoperative geriatric screening and assessment for surgical patients. **B** Perioperative geriatric support for surgical teams. *GA* Geriatric assessment, *dpt* department, *MDT* multidisciplinary team meeting, *RFN* reference nurses

Preoperative geriatric screening and geriatric assessment for surgical patients is summarised in Fig. 1A. Preoperative screening to identify patients with a geriatric risk profile is performed in 25 hospitals (46%). One or more of the following screening instruments are used: Flemish version of the Triage Risk Screening Tool (fTRST, *n* = 12), Identification of Seniors at Risk (ISAR, *n* = 7), Geriatric 8 (G8, *n* = 5), Edmonton Frail Scale (EFS, *n* = 2) and Short Emergency Geriatric Assessment (SEGA, *n* = 2). In 17 hospitals (32%), a positive screening is systematically followed by geriatric assessment. Geriatric assessment for surgical patients in an outpatient clinic is available in 25 hospitals (46%), of which 5 perform it systematically (i.e. according to a standardised procedure in a selected patient group) and 20 non-systematically. Geriatric assessment for surgical patients in the emergency department is available in 16 hospitals (30%), systematically in 6 and non-systematically in 10 hospitals. Geriatric assessment for surgical patients on the hospital ward is available in 30 hospitals (56%), systematically in 4 and non-systematically in 26 hospitals. Details per surgical specialty are summarised in Online Appendix 2.

Perioperative geriatric services for surgical teams are summarised in Fig. 1B (with details per surgical specialty in Online Appendix 2). Non-medical advice for surgical patients by geriatric teams is given in 49 hospitals (91%), of which 11 do not monitor adherence to their recommendations, 32 do provide follow-up, and 6 provide direct care themselves. Forty-four (82%) provide medical advice for surgical patients in their hospitals, of which 11 do not monitor adherence to their recommendations, 23 do provide follow-up, and 10 provide direct care themselves. In 26 hospitals (48%), geriatric teams participate in multidisciplinary team meetings for surgical patients: preoperatively (e.g. decision whether or not to operate) in 19 hospitals (35%) and postoperatively in 17 hospitals (32%). In 30 hospitals (56%), there are geriatric reference nurses on surgical wards, i.e. nurses within the surgical team that are trained or experienced in geriatric care and responsible for facilitating the identification and management of geriatric patients on their ward. Seven hospitals (13%) provide extensive protocols on the management of geriatric syndromes or geriatric problems in the perioperative period, 24 hospitals (44%) provide less extensive protocols, and 23 hospitals (43%) do not provide geriatric protocols for surgical teams. In 16 hospitals (30%), geriatric teams provide education and training for surgical teams: less than once per year in 6 hospitals, 1 to 3 times per year in 8 hospitals and more than 3 times per year in 2 hospitals.

**Fig. 2:**
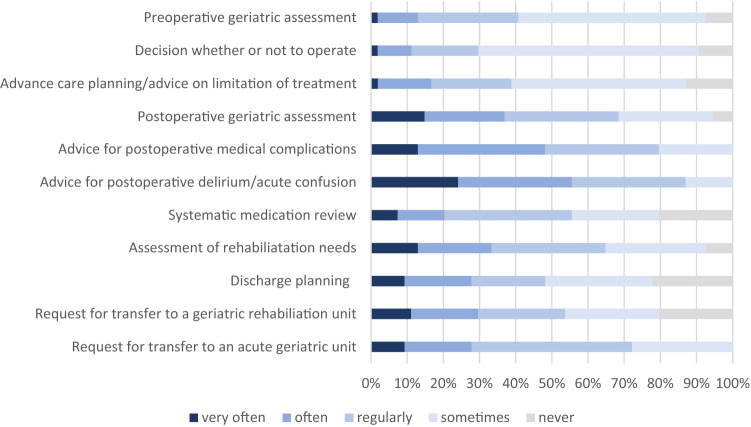
Time allocation of the geriatric team to different types of services for surgical patients and surgical teams

The time spent by geriatric teams on different types of services for surgical patients and surgical teams in a regular month is shown in Fig. 2. Activities most frequently performed (i.e. by more than one third of geriatric teams) are: (1) advice for postoperative delirium or acute confusion, (2) advice for postoperative medical complications, (3) postoperative geriatric assessment and (4) postoperative assessment of rehabilitation needs.

**Fig. 3:**
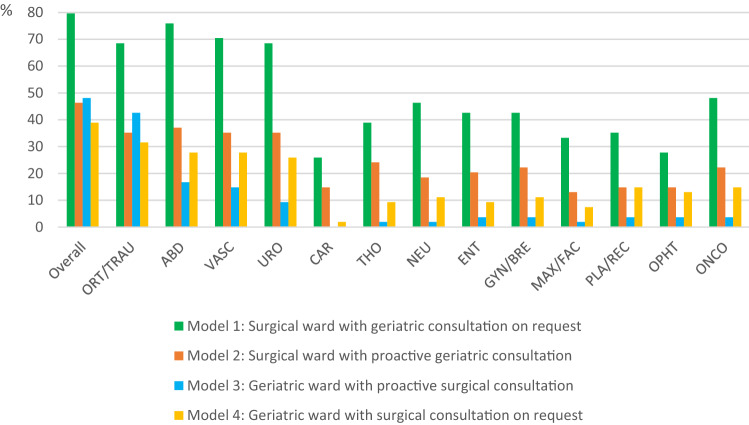
Geriatric-surgical care models and their distribution among surgical specialties. *ORT/TRAU* Orthopaedic/trauma surgery, *ABD* abdominal surgery, *VASC* vascular surgery, *URO* urologic surgery, *CAR* cardiac surgery, *THO* thoracic surgery, *NEU* neurosurgery, *ENT* ear–nose–throat surgery, *GYN/BRE* gynaecologic/breast surgery, *MAX/FAC* maxillofacial surgery, *PLA/REC* plastic/reconstructive surgery, *OPHT* ophthalmologic surgery, *ONCO* oncologic surgery

Finally, four geriatric-surgical care models during the perioperative hospitalisation were explored. The definitions of the care models and their distribution among surgical specialties are detailed in Fig. 3. In Model 1, the geriatric care is reactive, in model 2, 3 and 4 proactive. Model 2 and 3 are collaborative geriatric-surgical care models. Overall, 43 hospitals applied model 1 (80%), 25 applied model 2 (46%), 26 applied model 3 (48%), and 21 applied model 4 (39%). The different models overlap. Across surgical specialties, model 1 is the predominant perioperative care model, followed by model 2, except for orthopaedic/trauma surgery, where model 3 is the second most frequent model.

### Reflections on current practice and ideas for the future

The majority of respondents (98%) perceived a need to increase geriatric input for surgical patients in their hospital. When asked to rank surgical specialties according to their need for geriatric support, regardless of the support already given, the following top five emerged: (1) orthopaedic/trauma surgery, (2) vascular surgery, (3) abdominal surgery, (4) oncologic surgery and (5) urologic surgery. Online Appendix 3 gives an overview per surgical specialty of geriatric services currently provided in relation to the estimated need for geriatric support.

When asked about the best model for perioperative geriatric-surgical care, irrespective of current number of geriatricians or financial support, no one preferred model 1 as a single option. Nine respondents (17%) preferred model 2, 27 respondents (50%) preferred model 3, and 5 respondents (9%) preferred model 4. The 13 remaining respondents preferred a combination of different models.

Geriatricians who preferred hospitalisation on a surgical ward with proactive geriatric consultation (model 2) supported their choice for the surgical ward with the following statements:

- The geriatric team has insufficient expertise in perioperative care for the wide variety of surgical procedures.
- Immediate postoperative care is best provided on a surgical ward (until all surgical complications have been resolved).
- It is difficult to get a surgeon bedside once the surgical patient is on a geriatric ward.
- The geriatric team cannot take everything into its own hands and is already burdened enough.

Geriatricians who preferred hospitalising geriatric-surgical patients on a geriatric ward (model 3 or 4) mentioned the following reasons for their choice:

- It is difficult to implement geriatric care principles on a surgical ward.
- Hospitalisation on a geriatric ward guarantees a continuous and efficient holistic multidisciplinary approach.
- The geriatrician will know the patient best and is better positioned in the geriatric ward to coordinate the non-surgical care.
- The surgeon lacks time to address complex problems and can focus on an optimal surgical follow-up.
- It is the most manageable and satisfactory for the surgeon and geriatrician and the safest for the patient, provided that the geriatric team has sufficient surgical knowledge.
- The culture of multidisciplinary cooperation and shared decision making on geriatric wards leads to good quality decisions for the patient.

Geriatricians who preferred combining care models noted the following reasons:

- Fitter older patients or patients undergoing complex surgical procedures are best hospitalised on surgical wards, while frailer older patients or patients undergoing emergent surgical procedures are best hospitalised on geriatric wards.
- Patients should receive appropriate geriatric or surgical care on the ward where they are initially hospitalised. Patient transfers should be limited.

**Fig. 4:**
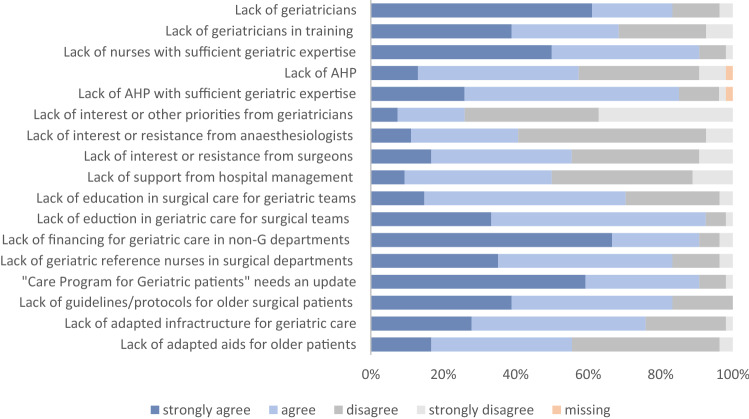
Perceived barriers to the development of geriatric-surgical services. *AHP* Allied health professionals, *G* geriatrics

Respondents were then asked to indicate the extent to which they agreed with a series of barriers to the further development of geriatric-surgical care in their hospital. They could also suggest additional barriers. The result is shown in Fig. 4. The main barriers were: (1) lack of financing for geriatric care in non-geriatrics departments, (2) lack of geriatricians, (3) current legislation, i.e. the Belgian ‘Care Program for Geriatric Patients’, needs an update and (4) lack of nurses with sufficient geriatric expertise.

Additional comments or suggestions regarding perioperative care for geriatric patients in Belgium, could be mapped according to three main themes: (1) geriatric screening and assessment, (2) education and training and (3) healthcare organisation.

Regarding geriatric screening and assessment, the following points were raised:

- Older surgical candidates should systematically receive preoperative geriatric risk screening by the non-geriatric team or the patient’s general practitioner.
- For elective patients, screening should be done in the preoperative outpatient clinic. For emergency admissions, screening should be done in the emergency department.
- Early screening can sensitise surgical teams to refer for ambulatory geriatric assessment in the geriatric day hospital or to involve the geriatric team early during hospitalisation for further geriatric assessment and interventions.
- When more complex surgery is planned in older patients, preoperative geriatric assessment should be mandatory (as for trans-catheter aortic valve procedures in Belgium).

Regarding education and training respondents noted:

- Surgeons, anaesthesiologists and their teams, as well as emergency department teams, should receive more education and training in geriatric care principles and risk/benefit of surgery in older patients. Geriatricians and their teams should receive more education and training regarding perioperative care.
- Education and training should start in medical school, through a geriatric surgery course and clinical internships, and should be encouraged after graduation, through postgraduate courses and by linking training in geriatric surgery to accreditation.
- Education and training for surgeons should encompass the holistic geriatric approach, prevention of common complications in older patients (e.g. cognitive, functional, nutritional), communication with primary care, and the importance of completeness of medical records and discharge letters (in terms of medical history, clinical examination, list of drugs on admission and discharge, and postoperative complications).

Suggestions on healthcare organisation can be summarised as follows:

- The hospital should have a clear business plan and well-developed care paths for geriatric-surgical patients. These care paths should, among others, define the roles of geriatricians and primary care physicians in the perioperative trajectory.
- It may be an idea to provide interconnected services in hospitals, such as side-by-side geriatric and surgical wards, as well as mixed geriatric-surgical wards.
- Provision of geriatric expertise and infrastructure in the emergency department should be ensured.
- A legal framework and adapted financing for collaborative services and shared responsibility should be established.
- The permitted length of hospital stay should be adapted to the complexity of the patient. There should be a mandatory multidisciplinary meeting for every patient with a prolonged hospital stay (e.g. longer than 1 week).

## Discussion

Our survey was designed to explore geriatric care for surgical patients in Belgian hospitals and geriatricians’ reflections on current practice. The data show that geriatric teams in Belgian hospitals offer a variety of geriatric services for surgical patients. However, these services are mainly reactive, i.e. at the request of the surgical team, and usually take place in the postoperative period. This is in contrast with emerging evidence that proactive and collaborative geriatric-surgical care leads to better outcomes compared to reactive care [15, 24, 25] Indeed, by providing reactive care, geriatric teams miss the opportunity of prevention, early detection and early treatment regarding geriatric syndromes and postoperative complications. Nevertheless, the survey showed several initiatives for more proactive services: for instance, 19% of hospitals systematically offer geriatric assessment for a selected group of surgical candidates, either in a preoperative outpatient clinic (9%) or in the emergency department (11%). In addition, there were several hospitals with a proactive geriatric consultation model on surgical wards, mostly in abdominal surgery, orthopaedic/trauma surgery, vascular surgery and urology. The survey also revealed that among all surgical specialties, orthopaedic/trauma patients are most often admitted to acute geriatric wards with proactive surgical consultation. However, despite evolving evidence for orthogeriatric co-management [26, 27], less than half of respondents seem to provide this collaborative geriatric-surgical care model. Importantly, almost all respondents feel that there is a need to increase geriatric input for surgical patients in their hospital. The greatest need was felt for orthopaedic/trauma surgery, vascular surgery and abdominal surgery. In current literature, studies in the orthogeriatric population predominate, but there seems to be an evolution towards scientific research in other surgical specialties [15]. However, a recent review showed that only few studies have compared proactive geriatric care with usual care in surgical specialties other than orthopaedic/trauma surgery [28].

Important to mention is that geriatric care is well implemented in Belgian hospitals due to a Royal Decree which states that hospitals must provide in acute geriatric hospital wards, an IGCT, a multidisciplinary geriatric day hospital, an outpatient clinic for geriatric patients and a partnership with primary care [10]. Our survey reveals implementation rates near 100% for these 5 components of the Belgian ‘Care Program for Geriatric Patients’. The ImAGE.eu survey and two surveys by the European Union Geriatric Medicine Society showed that this is not the case in many other European countries [29–31]. They showed that the recognition of geriatrics as a clinical specialty and implementation of geriatric care varies widely among European countries and that Belgium was one of the first countries to introduce acute geriatric hospital wards and to recognise geriatrics as a medical subspecialty. Comparing geriatric services for surgical patients with other European countries is difficult, as to date only in the UK a similar survey was conducted in 2013, with an update in 2017. The UK surveys showed similar results and a favourable evolution over time: reactive postoperative care remains predominant in the UK but has increased from 26 to 77% over time. Moreover, 14 newly established geriatrician-led preoperative clinics were documented between 2014 and 2017. In the UK, the Perioperative care of Older People undergoing Surgery (POPS) Special Interest Group from the British Geriatrics Society, launched in 2012, plays a leading role in promoting geriatric care for surgical patients. To our knowledge, the UK and Italy are the only European countries in which consensus recommendations for the perioperative management of older patients were published (in 2021 and 2020, respectively) [32, 33].

Although Belgium has an established national framework for geriatric care, it is notable that most respondents indicated unadjusted legislation and healthcare financing as main barriers to expand geriatric services for surgical patients. This can be explained by the fact that, for patients hospitalised on surgical wards, consultative IGCT-based care remains the recommended standard care model. Furthermore, referral to the geriatric day hospital requires a referral from the patients’ general practitioner to obtain financing for a multidisciplinary geriatric evaluation, which hinders direct referral from other medical specialties. Currently, there is no legal or financial framework in Belgium that supports collaborative care with shared responsibility between the surgical and the geriatric team during the perioperative hospitalisation. In addition, there is a reform of financing towards per-case payment for several surgical interventions, which may also lead to insufficient financing for the indispensable multidisciplinary management of older patients with a geriatric profile. Another important barrier to further expand proactive and collaborative geriatric services for surgical patients are shortages in the geriatric work force. Belgium encountered a cumulative shortfall of 119 graduates in geriatric medicine between 2004 and 2020, which is very high considering the total amount of 386 recognised geriatricians in 2020 [34, 35]. Shortage of geriatricians is an international phenomenon. In a recent survey in 22 countries half of respondents reported a lack of geriatricians in their country and most respondents suggested doubling current numbers [36]. Many countries are taking steps to recognise geriatrics as a clinical specialty or to encourage medical students to specialise in geriatrics, but this is a work in progress [29, 34, 36]. Apart from a lack of geriatricians, there is also a paucity of nurses with sufficient geriatric expertise. There is a need for more IGCT staff with co-management assignments and for geriatric reference nurses within non-geriatric teams to collaborate with. In 2007, the Belgian government mandated geriatric reference nurses on non-geriatric wards as clinical leaders responsible for the implementation of geriatric care within their team. However, this requirement was withdrawn from the Royal Decree in 2014. Nevertheless, according to our survey more than half of the participants still provide geriatric reference nurses on surgical wards. This may indicate that geriatric and surgical teams are convinced of their added value. Increasing the number of geriatric nurses represents a serious challenge, as currently there is a major workforce crisis across Europe which requires urgent action from politicians and healthcare managers [37]. Well-designed geriatric care pathways, teaching geriatric care principles in basic curricula and upskilling non-geriatric teams in the principles of geriatric medicine could partially overcome these shortages [36, 38, 39].

Although healthcare systems differ among countries, this survey can provide clinicians, hospital boards and policy makers all over Europe with useful information to reorganise perioperative care for frail older patients. The strength of this study is that, after the UK survey, this is the second initiative to map geriatric care for surgical patients in a European country. However, some methodological limitations of this study are to be mentioned. First, with a response rate of 59%, the survey does not display a complete picture of geriatric care for surgical patients across Belgium. The number of participants may have been influenced by the timing of the survey. Initial reminders by e-mail were sent during the summer holiday and final reminders by telephone were prematurely interrupted because of a new Covid-19 wave. Nevertheless, the final sample was diverse in terms of size and type of hospitals and geographic distribution. Second, there might have been response bias as respondents may provide more geriatric services for surgical patients in their hospital than non-respondents. Third, the survey was sent to heads of geriatrics departments, who are not necessarily directly involved in the daily care of surgical patients. On the other hand, they could ask for input from their team. Fourth, we did not survey the opinion of surgeons and anaesthesiologists and their teams.

Further research examining experience with geriatric support and perceived needs for support from the perspective of surgical teams is needed. In addition, future studies should focus on the clinical and cost-effectiveness of preoperative geriatric optimisation clinics and geriatric-surgical co-management during the perioperative hospitalisation, not only in the orthopaedic/trauma population but also in other surgical specialties. Indeed, gathering scientific evidence is key to obtaining recognition and financing for these geriatric services for surgical patients [3, 40]. To be able to upscale the studied interventions to clinical practice and to enhance sustainability after the implementation effort, it is important to incorporate implementation science methodology into intervention studies [41]. Geriatric societies with special interest groups on geriatric-surgical care, such as the American Geriatrics Society and the British Geriatrics Society, emphasise the importance of good scientific quality research in order to develop consensus on adequate care pathways and clinical guidelines for surgical patients with geriatric care needs. There is also a need for geriatric-specific quality and outcome indicators and for institutional and national registration and audits of these indicators to allow benchmarking and improvement of existing care programmes [32, 38, 42, 43].

In conclusion, geriatric care for surgical patients in Belgium is mainly reactive, but proactive services are emerging. The main barriers to improve geriatric-surgical care are a need to update current legislation and healthcare financing and to resolve staff shortages in the geriatric work field. Innovative care pathways and guidelines for geriatric-surgical care, supported by clinical research and national data collection to gather evidence on clinical and economic effectiveness, are indispensable and must be given priority.

## Supplementary Information

Below is the link to the electronic supplementary material.

Appendix 1: Survey questionnaire (translated in English). (DOCX 42 kb)

Appendix 2: Geriatric services delivered by geriatric teams per surgical specialty. (DOCX 210 kb)

Appendix 3: Geriatric services provided in relation to perceived need for geriatric input. (DOCX 17 kb)

## Data Availability

Available upon contacting the corresponding author.

## References

[1] Etzioni DA, Liu JH, Maggard MA, Ko CY (2003). The aging population and its impact on the surgery workforce. Ann Surg.

[2] Partridge JS, Harari D, Dhesi JK (2012). Frailty in the older surgical patient: a review. Age Ageing.

[3] Partridge JS, Harari D, Martin FC, Dhesi JK (2014). The impact of pre-operative comprehensive geriatric assessment on postoperative outcomes in older patients undergoing scheduled surgery: a systematic review. Anaesthesia.

[4] Beggs T, Sepehri A, Szwajcer A, Tangri N, Arora RC (2015). Frailty and perioperative outcomes: a narrative review. Can J Anaesth J Can D'anesth.

[5] Rubenstein LZ, Stuck AE, Siu AL, Wieland D (1991). Impacts of geriatric evaluation and management programs on defined outcomes: overview of the evidence. J Am Geriatrics Soc.

[6] Van Craen K, Braes T, Wellens N, Denhaerynck K, Flamaing J, Moons P (2010). The effectiveness of inpatient geriatric evaluation and management units: a systematic review and meta-analysis. J Am Geriatr Soc.

[7] Ellis G, Gardner M, Tsiachristas A, Langhorne P, Burke O, Harwood RH (2017). Comprehensive geriatric assessment for older adults admitted to hospital. Cochrane Datab Syst Rev.

[8] Fox MT, Persaud M, Maimets I, O'Brien K, Brooks D, Tregunno D (2012). Effectiveness of acute geriatric unit care using acute care for elders components: a systematic review and meta-analysis. J Am Geriatr Soc.

[9] Flood KL, Booth K, Vickers J, Simmons E, James DH, Biswal S (2018). Acute care for elders (ACE) team model of care: a clinical overview. Geriatrics.

[10] Federale Overheidsdienst Volksgezondheid Veiligheid van de voedselketen en Leefmilieu. Koninklijk besluit tot wijziging van het koninklijk besluit van 29 januari 2007 houdende vaststelling eensdeels, van de normen waaraan het zorgprogramma voor de geriatrische patiënt moet voldoen om te worden erkend en, anderdeels, van bijzondere aanvullende normen voor de erkenning van ziekenhuizen en ziekenhuisdiensten. 2014. https://etaamb.openjustice.be/nl/koninklijk-besluit-van-26-maart-2014_n2014024118. Accessed 12 Jan 2022

[11] Deschodt M, Claes V, Van Grootven B, Milisen K, Boland B, Flamaing J et al (2015) Report 245 from the Belgian healthcare knowledge center (KCE): comprehensive geriatric care in hospitals: the role of inpatient geriatric consultation teams. https://kce.fgov.be/nl/publicaties/alle-rapporten/globale-geriatrische-benadering-rol-van-de-interne-geriatrische-liaison-teams. Accessed 12 Jan 2022

[12] Deschodt M, Flamaing J, Haentjens P, Boonen S, Milisen K (2013). Impact of geriatric consultation teams on clinical outcome in acute hospitals: a systematic review and meta-analysis. BMC Med.

[13] Deschodt M, Jeuris A, Van Grootven B, Van Waerebeek E, Gantois E, Flamaing J (2020). Adherence to recommendations of inpatient geriatric consultation teams: a multicenter observational study. Eur Geriatr Med.

[14] Van Grootven B, Flamaing J, Dierckx de Casterle B, Dubois C, Fagard K, Herregods MC (2017). Effectiveness of in-hospital geriatric co-management: a systematic review and meta-analysis. Age Ageing.

[15] Van Grootven B, Mendelson DA, Deschodt M (2020). Impact of geriatric co-management programmes on outcomes in older surgical patients: update of recent evidence. Curr Opin Anaesthesiol.

[16] Alvarez-Nebreda ML, Bentov N, Urman RD, Setia S, Huang JC, Pfeifer K (2018). Recommendations for preoperative management of frailty from the society for perioperative assessment and quality improvement (SPAQI). J Clin Anesth.

[17] Partridge JSL, Aitken RM, Dhesi JK (2019). Perioperative medicine for older people: learning across continents. Australas J Ageing.

[18] National Guideline Centre (UK) (Aug 2020) Evidence review for preoperative optimisation clinics in older adults: perioperative care in adults: evidence review D. London: National Institute for health and care excellence (NICE) (NICE Guideline, No. 180). https://www.ncbi.nlm.nih.gov/books/NBK561976/. Accessed 12 Jan 2022 32931176

[19] Partridge JS, Collingridge G, Gordon AL, Martin FC, Harari D, Dhesi JK (2014). Where are we in perioperative medicine for older surgical patients? A UK survey of geriatric medicine delivered services in surgery. Age Ageing.

[20] Joughin AL, Partridge JSL, O'Halloran T, Dhesi JK (2019). Where are we now in perioperative medicine? Results from a repeated UK survey of geriatric medicine delivered services for older people. Age Ageing.

[21] Thillainadesan J, Hilmer S, Close J, Kearney L, Naganathan V (2019). Geriatric medicine services for older surgical patients in acute hospitals: results from a binational survey. Aust J Ageing.

[22] Federale Overheidsdienst Volksgezondheid Veiligheid van de voedselketen en Leefmilieu. Contact en erkenningsgegevens van de gezondheidsinstellingen in Vlaanderen, Brussel en Wallonie (2020). https://www.health.belgium.be/nl/gezondheid/organisatie-van-de-gezondheidszorg/delen-van-gezondheidsgegevens/gezondheidszorginstellingen. Accessed 12 Jan 2022.

[23] Kammerlander C, Roth T, Friedman SM, Suhm N, Luger TJ, Kammerlander-Knauer U, Blauth M (2010). Ortho-geriatric service—a literature review comparing different models. Osteoporosis Int.

[24] Eamer G, Taheri A, Chen SS, Daviduck Q, Chambers T, Shi X (2018). Comprehensive geriatric assessment for older people admitted to a surgical service. Cochrane Datab Syst Rev.

[25] Van Heghe A, Mordant G, Dupont J, Dejaeger M, Laurent MR, Gielen E (2022). Effects of orthogeriatric care models on outcomes of hip fracture patients: a systematic review and meta-analysis. Calcif Tissue Int.

[26] Pioli G, Bendini C, Pignedoli P, Giusti A, Marsh D (2018). Orthogeriatric co-management—managing frailty as well as fragility. Injury.

[27] Patel JN, Klein DS, Sreekumar S, Liporace FA, Yoon RS (2020). Outcomes in multidisciplinary team-based approach in geriatric hip fracture care: a systematic review. J Am Acad Orthop Surg.

[28] Thillainadesan J, Hilmer SN, Fleury AM, Naganathan V (2022). New horizons in the perioperative care of older adults. Age Ageing.

[29] Deschodt M, Boland B, Lund CM, Saks K, Velonaki VS, Samuelsson O (2018). Implementation of geriatric care models in Europe (imAGE.eu): a cross-sectional survey in eight countries. Eur Geriatr Med.

[30] Kolb G, Andersen-Ranberg K, Cruz-Jentoft A, O’Neill D, Topinkova E, Michel JP (2011). Geriatric care in Europe—the EUGMS survey part I: Belgium, Czech Republic, Denmark, Germany, Ireland, Spain, Switzerland. Eur Geriatr Med.

[31] Ekdahl A, Fiorini A, Maggi S, Pils K, Michel JP, Kolb G (2012). Geriatric care in Europe—the EUGMS survey part II: Malta Sweden Austria. Eur Geriatr Med.

[32] British Geriatrics Society (2021) Guideline for perioperative care for people living with frailty undergoing elective and emergency surgery. https://www.bgs.org.uk/sites/default/files/content/CPOC-BGS-Guideline%20for%20Perioperative%20Care%20for%20People%20Living%20with%20Frailty%20Undergoing%20Elective%20and%20Emergency%20Surgery.pdf. Accessed 12 Jan 2022 10.1093/ageing/afac237 36436009

[33] Aceto P, Antonelli Incalzi R, Bettelli G, Carron M, Chiumiento F, Corcione A (2020). Perioperative management of elderly patients (PriME): recommendations from an Italian intersociety consensus. Aging Clin Exp Res.

[34] Devos C, Cordon A, Lefèvre M, Obyn C, Renard F, Bouckaert N et al (2019) Report 313C from the Belgian healthcare knowledge center (KCE): performance of the Belgian health system. https://kce.fgov.be/sites/default/files/atoms/files/KCE_313C_Performance_Belgian_health_system_Report.pdf. Accessed 12 Jan 2022

[35] Cel planning van het Aanbod van de Gezondheidszorgberoepen. Jaarverslag van de planningscommissie—medisch aanbod (2020). https://overlegorganen.gezondheid.belgie.be/sites/default/files/documents/jaarverslag_2020_nl.pdf. Accessed 12 Jan 2022

[36] Pitkälä KH, Martin FC, Maggi S, Jyväkorpi SK, Strandberg TE (2018). Status of geriatrics in 22 Countries. J Nutr Health Aging.

[37] Michel JP, Ecarnot F (2020). The shortage of skilled workers in Europe: its impact on geriatric medicine. Eur Geriatr Med.

[38] McGory ML, Kao KK, Shekelle PG, Rubenstein LZ, Leonardi MJ, Parikh JA (2009). Developing quality indicators for elderly surgical patients. Ann Surg.

[39] Dhesi J, Moonesinghe SR, Partridge J (2019). Comprehensive geriatric assessment in the perioperative setting: Where next?. Age Ageing.

[40] Eamer G, Saravana-Bawan B, van der Westhuizen B, Chambers T, Ohinmaa A, Khadaroo RG (2017). Economic evaluations of comprehensive geriatric assessment in surgical patients: a systematic review. J Surg Res.

[41] Peters DH, Adam T, Alonge O, Agyepong IA, Tran N (2013). Implementation research: what it is and how to do it. BMJ.

[42] Van Grootven B, McNicoll L, Mendelson DA, Friedman SM, Fagard K, Milisen K (2018). Quality indicators for in-hospital geriatric co-management programmes: a systematic literature review and international Delphi study. BMJ Open.

[43] American College of Surgeons (2019) Geriatric surgery verification program standards. https://www.facs.org/quality-programs/geriatric-surgery/standards. Accessed 12 Jan 2022

