# Supplementary material for: Geriatric care for surgical patients: results and reflections from a cross-sectional survey in acute Belgian hospitals
Source: Eur Geriatr Med. 2023 Jan 24;14(2):239–49. doi: 10.1007/s41999-023-00748-3 (PMC9870777; doi:10.1007/s41999-023-00748-3)
Supplement: Supplementary file 1 — Appendix 1: Survey questionnaire (translated in English). (DOCX 42 kb) [file 41999_2023_748_MOESM1_ESM.docx]

**Appendix 1: Survey Questionnaire (translated in English)**

**Geriatric care for surgical patients in Belgian hospitals: a national survey.**

**PART 1: Geriatric services for surgical patients in the hospital**

- What **surgical specialties** are present in your hospital?
- Abdominal surgery
- Orthopaedic/trauma surgery
- Vascular surgery
- Urologic surgery
- Cardiac surgery
- Thoracic surgery
- Neurosurgery
- Ear-nose-throat surgery
- Gynaecologic/breast surgery
- Maxillofacial Surgery
- Plastic/reconstructive surgery
- Ophthalmologic surgery
- Oncologic surgery
- others: __
- Is **screening for a geriatric risk profile** performed preoperatively in older surgical patients?
  - No
  - Yes, for one or more surgical specialties

→ If yes, Please indicate for which surgical specialties:

(→ list of surgical specialties present in the hospital is displayed)

→ If yes, Which screening instrument(s)? __________________

→ If yes, Does risk screening systematically lead to a geriatric evaluation in high-risk patients?

- - - No, not systematically
    - Yes, a positive screening is always followed by a geriatric assessment
- Is a **preoperative geriatric assessment** performed in geriatric-surgical patients at the **outpatient clinic** as part of a standardised program (e.g. surgery clinic, anaesthesia clinic, geriatric day hospital)?

- No
- Yes, but not systematically***
- Yes, systematically*** for one or more surgical specialties

**systematically = according to a standardised procedure in a selected patient group*

→ If yes, Please indicate for which surgical specialties:

(→ list of surgical specialties present in the hospital is displayed)

- Is a **preoperative geriatric assessment** performed in geriatric-surgical patients in the **emergency department** as part of a standardised program?
- No
- Yes, but not systematically***
- Yes, systematically*** for one or more surgical specialties

**systematically = according to a standardised procedure in a selected patient group*

→ If yes, Please indicate for which surgical specialties:

(→ list of surgical specialties present in the hospital is displayed)

- Is a **preoperative geriatric assessment** performed in geriatric-surgical patients during **preoperative hospitalisation** as part of a standardised program?
- No
- Yes, but not systematically***
- Yes, systematically*** for one or more surgical specialties

**systematically = according to a standardised procedure in a selected patient group*

→ If yes, Please indicate for which surgical specialties:

(→ list of surgical specialties present in the hospital is displayed)

- Does the geriatric team participate in **multidisciplinary team meetings** for older surgical patients in the **preoperative** period (e.g. decision to operate or not)?
- No
- Yes, for one or more surgical specialties

→ If yes, Please indicate for which surgical specialties:

(→ list of surgical specialties present in the hospital is displayed

- Does the geriatric team participate in **multidisciplinary team meetings** for older surgical patients in the **postoperative** period (e.g. team meetings on surgical wards)?
- No
- Yes, for one or more surgical specialties

→ If yes, Please indicate for which surgical specialties:

(→ list of surgical specialties present in the hospital is displayed)

- Does the geriatric team provide a **education and training** for surgical teams regarding geriatric care?
- No, no specific education or training program is provided
- Yes

→ If yes, How often?

- less than once per year
- one to three times per year
- more than three times per year
- Are **geriatric reference nurses** provided on surgical wards?
- No
- Yes, for one or more surgical specialties

→ If yes, Please indicate for which surgical specialties:

(→ list of surgical specialties present in the hospital is displayed)

- Have **standardised protocols** been developed in your hospital for the management of geriatric syndromes/problems in surgical patients in the perioperative period?
- No
- Yes, but not yet standardised for all geriatric syndromes/problems
- Yes, comprehensive standardised protocols
- Does the geriatric team provide **medical advice** for surgical patients during the perioperative hospitalisation?
- No
- Yes

→ If yes, Please specify:

- advice only, whether or not the advice is implemented is not monitored
- advice with follow-up and encouraging the surgical team to implement the advice
- more than advice, the geriatric team implements all or part of the advice itself
- Does the geriatric team provide **non-medical advice** for surgical patients during the perioperative hospitalisation?
- No
- Yes

→ If yes, Please specify:

- advice only, whether or not the advice is implemented is not monitored
- advice with follow-up and encouraging the surgical team to implement the advice
- more than advice, the geriatric team implements all or part of the advice itself
- Indicate how often (in a typical month) your geriatric team performs the actions below for surgical patients (in general, regardless of surgical specialty).

| Preoperative geriatric assessment | never | sometimes | regularly | often | very often |
| --- | --- | --- | --- | --- | --- |
| Decision whether or not to operate | never | sometimes | regularly | often | very often |
| Early care planning or advice on therapy restriction | never | sometimes | regularly | often | very often |
| Postoperative geriatric assessment | never | sometimes | regularly | often | very often |
| Advice for postoperative medical complications | never | sometimes | regularly | often | very often |
| Advice for postoperative delirium/acute confusion | never | sometimes | regularly | often | very often |
| Systematic medication review | never | sometimes | regularly | often | very often |
| Assessment of rehabilitation needs | never | sometimes | regularly | often | very often |
| Discharge planning | never | sometimes | regularly | often | very often |
| Request for transfer to a geriatric rehabilitation unit | never | sometimes | regularly | often | very often |
| Request for transfer to an acute geriatric unit | never | sometimes | regularly | often | very often |

- Read the following 4 geriatric-surgical care models and indicate for which surgical specialties the geriatric-surgical care model is used in your hospital during the perioperative stay.

**Care model 1: Surgical ward with geriatric consultation on request.**

The patient is hospitalised on a surgical ward and supervised the surgical team. The internal geriatric consultation team is requested for specific problems if deemed useful by the surgical team.

- This model is not applied in our hospital
- This model is applied in our hospital for one or more surgical specialties

→ If yes, Please indicate for which surgical specialties:

(→ list of surgical specialties present in the hospital is displayed)

**Care model 2: Surgical ward with proactive geriatric consultation**

The patient is hospitalised on a surgical ward and supervised by the surgical team. The geriatric team performs 'case finding' or performs systematic geriatric consultations from admission to discharge or attends multidisciplinary team meetings.

- This model is not applied in our hospital
- This model is applied in our hospital for one or more surgical specialties

→ If yes, Please indicate for which surgical specialties:

(→ list of surgical specialties present in the hospital is displayed)

**Care model 3: Geriatric ward with proactive surgical consultation**

The patient is hospitalised on a geriatric ward and supervised by the geriatric team. The surgical team visits the patient systematically and patients are systematically discussed between teams (shared responsibility).

- This model is not applied in our hospital
- This model is applied in our hospital for one or more surgical specialties

→ If yes, Please indicate for which surgical specialties:

(→ list of surgical specialties present in the hospital is displayed)

**Care model 4: Geriatric ward with surgical consultation on request**

The patient is hospitalised on a geriatric ward and supervised by the geriatric team. The surgeon has a consultative role.

- This model is not applied in our hospital
- This model is applied in our hospital for one or more surgical specialties

→ If yes, Please indicate for which surgical specialties:

(→ list of surgical specialties present in the hospital is displayed)

**PART 2: Reflections on current practice and ideas for the future**

- In your opinion, what is the **preferred model** for perioperative geriatric-surgical care (regardless of the current number of geriatricians or available financial resources)?***

**Indicating multiple care models is possible if you prefer a combination of care models*

- Care model 1: Surgical ward with geriatric consultation on request
- Care model 2: Surgical ward with proactive geriatric consultation
- Care model 3: Geriatric ward with proactive surgical consultation and shared responsibility
- Care model 4: Geriatric ward with surgical consultation on request

Why? ____________

- Thinking about your hospital and the services you already provide, is there a **need to increase geriatric input** for older surgical patients in your hospital?
- Yes
- No
- In your opinion, to what extent do the following surgical specialties **need geriatric support** (regardless of geriatric support already provided)? *Rate on a scale of 1 to 10: 1 = very little and 10 = very much*

| Abdominal surgery | 1 | 2 | 3 | 4 | 5 | 6 | 7 | 8 | 9 | 10 |
| --- | --- | --- | --- | --- | --- | --- | --- | --- | --- | --- |
| Orthopaedic/trauma surgery | 1 | 2 | 3 | 4 | 5 | 6 | 7 | 8 | 9 | 10 |
| Vascular surgery | 1 | 2 | 3 | 4 | 5 | 6 | 7 | 8 | 9 | 10 |
| Urologic surgery | 1 | 2 | 3 | 4 | 5 | 6 | 7 | 8 | 9 | 10 |
| Cardiac surgery | 1 | 2 | 3 | 4 | 5 | 6 | 7 | 8 | 9 | 10 |
| Thoracic surgery | 1 | 2 | 3 | 4 | 5 | 6 | 7 | 8 | 9 | 10 |
| Neurosurgery | 1 | 2 | 3 | 4 | 5 | 6 | 7 | 8 | 9 | 10 |
| Ear-nose-throat surgery | 1 | 2 | 3 | 4 | 5 | 6 | 7 | 8 | 9 | 10 |
| Gynaecologic/breast surgery | 1 | 2 | 3 | 4 | 5 | 6 | 7 | 8 | 9 | 10 |
| Maxillofacial Surgery | 1 | 2 | 3 | 4 | 5 | 6 | 7 | 8 | 9 | 10 |
| Plastic/reconstructive surgery | 1 | 2 | 3 | 4 | 5 | 6 | 7 | 8 | 9 | 10 |
| Ophthalmologic surgery | 1 | 2 | 3 | 4 | 5 | 6 | 7 | 8 | 9 | 10 |
| Oncologic surgery | 1 | 2 | 3 | 4 | 5 | 6 | 7 | 8 | 9 | 10 |

- To what extent do you experience the following as a **barrier** to the development of geriatric-surgical care in your hospital?

| Lack of geriatricians | Strongly disagree | Disagree | Agree | Strongly agree |
| --- | --- | --- | --- | --- |
| Lack of geriatricians in training | Strongly disagree | Disagree | Agree | Strongly agree |
| Lack of nurses with sufficient geriatric expertise | Strongly disagree | Disagree | Agree | Strongly agree |
| Lack of allied health professionals | Strongly disagree | Disagree | Agree | Strongly agree |
| Lack of allied health professionals with sufficient geriatric expertise | Strongly disagree | Disagree | Agree | Strongly agree |
| Lack of interest or other priorities from geriatricians | Strongly disagree | Disagree | Agree | Strongly agree |
| Lack of interest or resistance from anaesthesiologists | Strongly disagree | Disagree | Agree | Strongly agree |
| Lack of interest or resistance from surgeons | Strongly disagree | Disagree | Agree | Strongly agree |
| Lack of support from hospital management | Strongly disagree | Disagree | Agree | Strongly agree |
| Lack of education/training in perioperative medicine for geriatric teams | Strongly disagree | Disagree | Agree | Strongly agree |
| Lack of education/training in geriatric care for surgical teams | Strongly disagree | Disagree | Agree | Strongly agree |
| Lack of financing for geriatric care on non-geriatric wards | Strongly disagree | Disagree | Agree | Strongly agree |
| Lack of reference nurses with training in geriatric care aspects on surgical wards | Strongly disagree | Disagree | Agree | Strongly agree |
| Current 'Care programme for the geriatric patient' (RD 2007-2014) needs an update in this respect | Strongly disagree | Disagree | Agree | Strongly agree |
| Lack of clear clinical guidelines/protocols for older surgical patients | Strongly disagree | Disagree | Agree | Strongly agree |
| Lack of adapted infrastructure for geriatric care | Strongly disagree | Disagree | Agree | Strongly agree |
| Lack of adapted aids for older patients | Strongly disagree | Disagree | Agree | Strongly agree |

- Are there **other** issues you consider a barrier? _____
- Do you have any **additional comments or suggestions** regarding perioperative care for geriatric patients in Belgium? _____

**PART 3: General information about the hospital and the geriatrics department**

- What is the name of your hospital? (*confidential*) ___
- How many campuses does your hospital consist of? ___ *(complete the table -> 1 row per campus)*

| Name of the campus  (confidential) | Number of operational G-beds | Components of the geriatric care program *(by campus)* |
| --- | --- | --- |
| _____ | _____ | - Acute geriatric hospital ward - Geriatric day hospital - Internal geriatric consultation team - Outpatient geriatric clinic - External liaison service |
| … |  |  |

- How many full-time equivalent (FTE) geriatricians currently work in your hospital's geriatrics department? ___
- How many full-time equivalent (FTE) trainees in internal, geriatric, or general medicine currently work in your hospital's geriatrics department? ___
- How many full-time equivalent (FTE) hospitalists (non-geriatricians or physicians in training) currently work in your hospital's geriatrics department? ___
- How many full-time equivalent (FTE) nurses and allied health professionals currently work in your internal geriatric consultation team? ___
- Is there currently an advanced practice nurse in geriatric care*** working in your hospital?

**A master's degree trained nurse responsible for hospital-wide innovative geriatric care*

- Yes
- No
