# Supplementary material for: Geriatric care for surgical patients: results and reflections from a cross-sectional survey in acute Belgian hospitals
Source: Eur Geriatr Med. 2023 Jan 24;14(2):239–49. doi: 10.1007/s41999-023-00748-3 (PMC9870777; doi:10.1007/s41999-023-00748-3)
Supplement: Supplementary file 2 — Appendix 2: Geriatric services delivered by geriatric teams per surgical specialty. (DOCX 210 kb) [file 41999_2023_748_MOESM2_ESM.docx]

LEGEND: GA: geriatric assessment; ED: emergency department; MDT: multidisciplinary team meeting.
