# Supplementary material for: Geriatric care for surgical patients: results and reflections from a cross-sectional survey in acute Belgian hospitals
Source: Eur Geriatr Med. 2023 Jan 24;14(2):239–49. doi: 10.1007/s41999-023-00748-3 (PMC9870777; doi:10.1007/s41999-023-00748-3)
Supplement: Supplementary file 3 — Appendix 3: Geriatric services provided in relation to perceived need for geriatric input. (DOCX 17 kb) [file 41999_2023_748_MOESM3_ESM.docx]

**Appendix 3: Geriatric services provided in relation to perceived need for geriatric input**

| **Surgical services in order of perceived need for**  **geriatric input**  (descending order) | N^a^ | **Geriatric services provided** | | | | | | |
| --- | --- | --- | --- | --- | --- | --- | --- | --- |
|  |  | Preoperative  Screening  n (%) | Preoperative GA | | | MDT | | Geriatrics reference nurses  on surgical ward  n (%) |
|  |  |  | Ambulatory  n (%) | ED  n (%) | Hospital ward  n (%) | Preoperative  n (%) | Postoperative  n (%) |  |
| 1. ORT/TRAU | 54 | 19 (35) | 5 (9) | 6 (11) | 4 (7) | 13 (24) | 16 (30) | 29 (54) |
| 1. VASC | 54 | 14 (26) | 0 (0) | 3 (6) | 2 (4) | 4 (7) | 6 (11) | 25 (46) |
| 1. ABD | 54 | 15 (28) | 0 (0) | 3 (6) | 2 (4) | 4 (7) | 6 (11) | 26 (48) |
| 1. ONCO | 44 | 18 (41) | 2 (5) | 1 (2) | 1 (2) | 13 (30) | 7 (16) | 17 (39) |
| 1. URO | 54 | 15 (28) | 2 (4) | 3 (6) | 2 (4) | 3 (6) | 5 (9) | 26 (48) |
| 1. CAR | 21 | 12 (57) | 2 (10) | 0 (0) | 1 (5) | 7 (33) | 4 (19) | 9 (43) |
| 1. NEU | 38 | 10 (26) | 0 (0) | 2 (5) | 1 (3) | 3 (8) | 3 (8) | 18 (47) |
| 1. THO | 41 | 9 (22) | 0 (0) | 1 (2) | 1 (2) | 2 (5) | 4 (10) | 20 (49) |
| 1. GYN/BRE | 49 | 11 (23) | 1 (2) | 3 (6) | 2 (4) | 4 (8) | 3 (6) | 19 (39) |
| 1. ENT | 53 | 9 (17) | 0 (0) | 3 (6) | 2 (4) | 2 (4) | 2 (4) | 17 (32) |
| 1. PLA/REC | 48 | 9 (19) | 0 (0) | 3 (6) | 2 (4) | 3 (6) | 2 (4) | 13 (27) |
| 1. MAX/FAC | 44 | 9 (21) | 1 (2) | 2 (5) | 1 (2) | 3 (7) | 2 (5) | 14 (32) |
| 1. OPHT | 51 | 8 (16) | 0 (0) | 3 (6) | 2 (4) | 2 (4) | 2 (4) | 15 (29) |
| LEGEND: ABD: abdominal surgery; CAR: cardiac surgery; ED: in the emergency department; ENT: ear-nose-throat surgery; GA: geriatric assessment; GYN/BRE: gynaecologic/breast surgery; MAX/FAC: maxillofacial surgery; MDT: multidisciplinary team meeting; n: number; NEU: neurosurgery; ONCO: oncologic surgery; OPHT: ophthalmologic surgery; ORT/TRAU: orthopaedic/trauma surgery; PLA/REC: plastic/reconstructive surgery; THO: thoracic surgery; URO: urologic surgery; VASC: vascular surgery; ^a^number of hospitals providing this surgical specialty (percentages calculated based on this number) | | | | | | | | |
